# Intergenerational education and premature mortality: a registry population-based study

**DOI:** 10.1093/eurpub/ckac129.504

**Published:** 2022-10-25

**Authors:** C Carmeli, D Anker, A Chiolero, S Cullati

**Affiliations:** 1 Population Health Laboratory, University of Fribourg, Fribourg, Switzerland; 2 Institute of Primary Health Care, University of Bern, Bern, Switzerland; 3 School of Population and Global Health, McGill University, Montreal, Canada; 4 Department of Readaptation and Geriatrics, University of Geneva, Geneva, Switzerland

## Abstract

Inequalities in premature mortality due to individual's attained education are well documented. Intergenerational educational trajectories, whereby both parental and individual education may affect mortality, have received less attention. Our aim was to assess the effect of intergenerational educational trajectories on sex- and cause-specific risks of chronic disease-related premature mortality in Switzerland. Data were from 695,972 individuals born between 1971 and 1980, who were followed from adolescence (10-19y) to mid-life (38-47y) in the Swiss National Cohort, a registry population-based study. Educational trajectories were categorized into four levels: High-High, High-Low, Low-High, Low-Low, which corresponded to the sequence of parental-individual attained education (exposure). Cause of death categories were cardiovascular disease (CVD), cancer, substance use and all other chronic diseases (outcome). We implemented a counterfactual-based framework to quantify inequalities and ran negative outcome controls to triangulate findings. Overall, inequalities were negligible for women and substantial for men, particularly in CVD and substance use deaths. Specifically, inequalities in CVD were negligible by age 30, while by age 45 inequalities due to a High-Low or Low-Low trajectory corresponded to 229 (95% confidence interval (CI): 99, 381) additional CVD deaths per 100,000 persons compared to a High-High trajectory. Inequalities in substance use deaths due to a High-Low trajectory corresponded to 106 (95% CI: 47, 208) additional deaths per 100,000 persons compared to a High-High trajectory by age 30, and increased afterwards. We identified sex- and cause-specific groups at high-risk of premature mortality, and life periods when inequalities due to both parental and individual education arise. Prioritization of prevention strategies in those life periods and groups may help reduce educational inequalities in chronic disease-related premature mortality.

